# Prognostic factor research: why it matters in orthopaedics and how we do it better

**DOI:** 10.1302/2633-1462.75.BJO-2025-0385.R1

**Published:** 2026-05-07

**Authors:** Zaid A. Hamoodi, Richard D. Riley, Gary S. Collins, Lianne Kearsley-Fleet, Jamie C. Sergeant, Adam C. Watts

**Affiliations:** 1 Centre for Epidemiology Versus Arthritis, Centre for Musculoskeletal Research, University of Manchester, Manchester Academic Health Science Centre, Manchester, UK; 2 Upper Limb Unit, Wrightington Hospital, Wrightington, Wigan and Leigh Teaching Hospitals NHS Foundation Trust, Wigan, UK; 3 Department of Applied Health Sciences, School of Health Sciences, College of Medicine and Health, University of Birmingham, Birmingham, UK; 4 National Institute for Health and Care Research (NIHR) Birmingham Biomedical Research Centre, Birmingham, UK; 5 Centre for Statistics in Medicine, Nuffield Department of Orthopaedics, Rheumatology and Musculoskeletal Sciences, University of Oxford, Oxford, UK; 6 Centre for Biostatistics, School of Health Sciences, Faculty of Biology, Medicine and Health, University of Manchester, Manchester Academic Health Science Centre, Manchester, UK; 7 Faculty of Health and Social Care Medicine, Edge Hill University, Ormskirk, UK

**Keywords:** Prognosis research, Prognostic factors, Prognosis, clinicians, trauma, orthopaedic research, arthroplasty surgery, statistical analysis, revision surgery, comorbidities, strength, patient-reported outcomes

## Abstract

By studying and predicting health outcomes, prognosis research can inform joint decision-making between a patient and clinician, and help to improve future health and care. The scope of prognosis research is broad. It can describe the natural history of a condition; identify prognostic factors (variables associated with future outcomes); develop and validate prediction models for estimating an individual’s outcome risk; and identify intervention effects that vary by specified individual characteristics. Research on prognostic factors enables personalized treatment, as it helps identify patient risk profiles that guide clinical decisions, trial design, and resource allocation, and it is a stepping stone into developing prognostic models for individualized risk predictions. However, it is imperative for prognosis research to have high standards of study design, analysis, and reporting. Inadequately executed research can lead to misleading prognostic information, resulting in inappropriate treatment or advice for some patients. A better understanding of the fundamentals of modern prognosis research methods can empower clinicians to more accurately appraise research findings in this field. This will lead to the identification and implementation of high-quality prognostic research findings, to improve healthcare and patient outcomes. However, prognostic research in orthopaedics often exhibits poor methodology, inconsistent reporting, and over-reliance on statistical significance. Recent advances in methodological guidance have provided important recommendations for study design, analysis, and reporting. Accurate interpretation requires adjusted analyses that consider known prognostic factors to prevent misleading conclusions. Key components include study registration, sample size planning, clear protocols and transparent reporting. Following these principles can greatly enhance the quality and clinical relevance of trauma and orthopaedic research. This overview introduces types of prognosis studies and then focuses on prognostic factor research and its importance within the field of Trauma and Orthopaedics (T&O). It outlines the latest advances, identifies current limitations, and directs readers towards best practices that should be adopted by researchers.

Cite this article: *Bone Jt Open* 2026;7(5):613–618.

## Introduction

In everyday practice, Trauma and Orthopaedic (T&O) surgeons are dedicated to providing the best care for patients. This includes consideration of a patient’s prognosis, which refers to understanding and predicting their likely outcomes to inform patient counselling, treatment decisions, and monitoring strategies.^1^ Without high-quality prognosis research findings, surgeons are restricted to their own professional experience or limited clinical data, which could lead to reliance on heuristics and risks cognitive bias.^2^ Consider an 80-year-old female who has been referred to the orthopaedic clinic due to painful advanced hip osteoarthritis. She wants to know about the natural history (typical prognosis) of patients with her condition, what her outcomes are likely to be given her age and other characteristics, the treatment option that is most likely to relieve her pain with the lowest risk of adverse events, as well as the likely outcome of any intervention and how long it will last.^3^ The clinician might be able to describe the likely prognosis for the whole population, but the patient might reasonably want to know what the likely outcome is for someone like her. What if we could use personal information, for example age, sex, BMI, smoking history, symptom severity, or prior surgical history, to help identify the likely prognosis for each individual, to better inform their treatment decisions? By predicting patients’ condition trajectories and likely outcomes, prognosis research has been essential in shaping treatment decisions across healthcare disciplines.^1^

Prognosis research is frequently discussed in terms of complications and side effects, rather than the fuller consideration of likely outcomes for patients who have the condition of interest.^4^ In 2013, a group of healthcare professionals, researchers, and journal editors proposed the PROGnosis RESearch Strategy (PROGRESS) framework, which consists of four different, but inter-related, types of prognosis studies:

- Type I (overall prognosis research): summarizing the overall (average) outcome value or risk among a group of individuals with a particular disease or health condition.^1^
- Type II (prognostic factor research): Identifying specific factors (variables) associated with changes in the outcome value or risk across individuals.^5^
- Type III (prognostic model research): Developing, validating, and evaluating the impact of prediction models to estimate an individual’s value or risk of a future outcome.^6^
- Type IV: (predictors of treatment effect): Identifying prognostic information to help tailor treatment decisions to an individual or group of individuals with similar characteristics.^7^

In T&O research, there is a wealth of research focused on investigating outcomes following joint arthroplasty, and particularly on failure. We will use this as an example to illustrate the various types of prognosis research. The start point (time for prognostication) is prior to arthroplasty surgery and the endpoint (future outcome of interest) is failure requiring revision surgery:

- Type I studies summarize the proportion of joint arthroplasty surgeries that require a revision within some time (e.g. two, five, or ten years). This information can be used for overall planning of the required resources to address these revisions. Knowing the typical course of a disease also allows clinicians and patients to weigh the benefits and burdens of treatments more effectively and to set realistic patient expectations.
- Type II studies identify prognostic factors: those variables associated with the failure of a joint arthroplasty (e.g. the association between age and the failure of a joint arthroplasty). This research is the first step towards individual outcome predictions, identifying potential sub-groups with increased risk of failure. This information can be used to inform decisions regarding the most appropriate treatments, and for planning services based on the burden of failed arthroplasties while considering the characteristics of the patient population. Prognostic factor research can also inform further research by identifying potential causal factors associated with failure of joint arthroplasties or factors that can interact with the treatment effectiveness.
- Type III studies focus on the development, validation, and impact assessment of a prognostic model that would estimate the individualized risk of failure for each patient considering a joint arthroplasty. By providing tailored prognostic estimates, such models could guide shared clinical decision-making between patients and clinicians, supporting discussions around the appropriateness, timing, or type of surgical intervention. Ultimately, they help align treatment plans with patient values, preferences, and risk profiles, leading to more informed and individualized care.
- Type IV studies focus on stratifying treatment choices based on prognostic information relevant to specific patient sub-groups and their expected responses to those treatments (i.e. understanding treatment effect heterogeneity). This information can be used in tailoring treatments for individual patients, thereby maximizing benefits and minimizing harms. For instance, it could inform the decision-making process regarding the necessity of proceeding with arthroplasty surgery. Additionally, it could assist in selecting the most suitable joint arthroplasty options, including fixation methods (cemented or uncemented), the material used, or the different bearing surfaces. This could be used to optimize patient outcomes, reduce unwanted effects, and increase the efficiency of healthcare.

All types of prognosis research can be used in informing and improving healthcare. This article focuses on prognostic factor research (Type II), which is a common type of prognosis research in T&O, but, like prognostic factor research in other fields, methodological reviews have identified limitations in the published literature.^8-10^ For example, a systematic review of prognostic factors in total elbow arthroplasty identified widespread methodological flaws, and found the quality of evidence to be low or very low.^4^ Therefore, in this article we aim to raise awareness of the fundamentals of prognostic factor research and introduce readers to what constitutes current best practice in this field. We will define what prognostic factors are, describe common limitations of existing prognostic factor studies, summarize recommendations for those undertaking or appraising prognostic factor research, and outline recent advances in this field.

## Prognostic factor research

A prognostic factor is any variable associated with the risk of a health outcome among individuals with a particular health condition.^11^ Prognostic factor research aims to identify prognostic factors by examining the strength of prognostic relationships between particular factors and future outcomes of interest.^5^ In T&O, prognostic factors for outcomes such as clinical endpoints and patient-reported outcomes, may include patient-related variables such as age, occupation, and disease severity, as well as treatment-related variables such as the type of non-surgical management or type of implant used.^12^ Prognostic factors may also include the characteristics of service providers, such as the level of experience of the clinician with the type of surgery, or the type of hospital where the treatment is administered.^12^ Also, in the last decade, biomarkers and genetics have been investigated as potential prognostic factors to guide management.^13,14^ Some of these markers, such as lactate and pH levels as prognostic factors associated with complications in polytrauma patients, are being used to guide clinical management.^15^

Prognostic factor studies can be classified into two types: exploratory and confirmatory studies.^5^ Exploratory studies aim to discover novel prognostic factors and produce initial evidence to support further investigation. Confirmatory (replication) studies seek to evaluate previously identified prognostic factors (i.e. those in exploratory studies) and to confirm their prognostic value over established prognostic factors.^5^ Confirmatory studies may fail to support the results of exploratory studies, indicating that the initial finding is likely to have been reached by chance, assuming that both have been conducted appropriately.^5^ In this way, both exploratory and confirmatory studies have an important role in prognostic factor research.

In the medical literature, many terms are used to describe prognostic factors, including prognostic variables, prognostic indicators, predictors, and prognostic determinants. The term risk factor is sometimes used to describe prognostic variables; however, it is an ambiguous term as it could be interpreted as implying a causal relationship between the risk factor and the outcome and hence is best avoided in this setting. A prognostic factor is associated with the development of an outcome in patients with a condition and does not necessarily need to be causal.^16^ Indeed, most prognostic factors are not causal (though, all causal factors of future outcomes are prognostic factors). To address this inconsistency, the PROGRESS partnership advocates the use of the term ‘prognostic factor’ in this field of research.

## The importance of prognostic factor research

Prognostic factor research plays an important role in guiding clinical and therapeutic decisions by enabling careful selection of (groups of) patients for specific treatments based on their absolute risk.^5^ Moreover, by having comprehensive information about prognostic factors, patients can be better informed about their condition, supporting more personalized and appropriate treatment decisions. For example, morbid obesity is reported to be associated with an increased risk of complications and the need for revision surgery following total knee arthroplasty.^17^ Therefore, it is important for patients to be informed about these risks, and they should be considered carefully before proceeding with the surgery.

Prognostic factor research is crucial for shaping the future of healthcare interventions and clinical trial design.^18,19^ For example, randomized trials might want to stratify for prognostic factors during randomization and analyses approaches, to help improve baseline balance and produce more efficient treatment effect estimates (minimization). Prognostic factors may motivate intervention research, for example through mechanisms to modify the value of a prognostic factor under the assumption that it is causal. Further, some prognostic factors may interact with treatment effects (i.e. some interventions may work well for some patients and not others), which motivates PROGRESS Type IV research.^19^

Regardless of their relationships to interventions, prognostic factors can be used in combination to develop prognostic models to estimate individual risk, enabling more tailored clinical decisions and information for each patient.^6^

Understanding prognostic factors also allows for more efficient allocation of healthcare resources and service planning.^20^ In joint arthroplasty surgery this knowledge can guide the selection of implants, for example the use of Latitude Legacy implant has been found to be associated with an increased hazard of failure following total elbow arthroplasty surgery, and help determine the optimal procedure volume,^21^ ultimately reducing complications and cutting costs for healthcare systems.^22^

## Limitations in prognostic factor research

Methodological limitations identified in prognostic factor research across different clinical areas include inconsistent terminology, unclear conceptualization, poor study design and statistical analysis (e.g. dichotomization of continuous variables or a lack of assessment of a factor’s added prognostic value over existing factors), incomplete or selective reporting, and a lack of confirmatory studies.^11^ These deficiencies have led to significant problems such as underpowered studies, retrospective studies of low quality, false-positive findings, publication bias, and research findings limited by data-dredging.^11,23^ Over-reliance on statistical significance, rather than the strength of the relationship between a prognostic factor and an outcome and the clinical importance of the results can also lead to misinterpretation.^10^

To effectively incorporate prognostic factor research into clinical patient management, it is vital to thoroughly evaluate and summarize all the available evidence.^24^ Well-conducted prognostic factor studies can significantly enhance our understanding of treatment outcomes and provide accurate information to support patient choices. While many exploratory studies in T&O have proposed valuable prognostic factors, these findings are often not confirmed or replicated. Future research should focus on replicating and validating current findings regarding prognostic factors. Additionally, addressing the variability among studies, such as differences in measurement methods and statistical analysis, will help facilitate meaningful meta-analyses and ultimately improve patient care.^5,12^ Furthermore, the search for novel prognostic factors should investigate added value beyond established prognostic factors, as many current factors (e.g. age) are easily measurable. Therefore, new factors must demonstrate prognostic information that exceeds these.

## Recommendations in prognostic factor research

Over the past two decades, there has been significant progress in the provision of methodological guidance for prognosis research, marked by the publication of recommendations and guidelines. These encompass the definitions used in prognosis research, study design, management of risk of bias, guidance on statistical analysis, and reporting guidelines.^1,5-7,25,26^ This section will provide an overview of some of these recommendations, which can be implemented in T&O research. For those intending to engage in prognostic factor research, it is advisable to explore the valuable resources, recommendations, and research guidelines available on the PROGRESS website.^27^

### Study protocol

When undertaking a prognostic factor study, researchers should understand where their study fits within the literature, if it is exploring new factors or confirming the prognostic value of known factors. It is recommended that the researchers publish a protocol and register the study to improve the transparency of reporting and avoid any possibility of data dredging.^28^ The definitions of prognostic factors, start points, endpoints (outcomes), and how they are all measured (using validated measurements if possible) should be clearly described.^11,23^

### Study type

Prospective cohort studies are preferable for prognostic research as they allow for clear inclusion criteria, standardization of measurements, and more complete baseline and follow-up data.^5^ Prospective studies, however, can be time-consuming and costly. Large healthcare databases, such as joint registries, can be used as data sources in prognostic factor research. However, researchers should take into account what limitations a large dataset might have (e.g. lack of standardization in measurements or lack of important prognostic factors to adjust for) and try to enhance the quality of the data, for example by linking data sources.^5,11,23^

### Statistical analysis

In statistical terms, it is crucial to report the strength of the relationship between investigated prognostic factors and the outcome of interest, using appropriate measures such as risk ratios, odds ratios, or hazard ratios.^29^ It is also important to always report the precision of those estimates using CIs.^29^

The statistical analyses in prognostic factor research can be broadly categorized into unadjusted and adjusted analyses. Unadjusted analysis examines the crude prognostic association between one factor and an outcome (e.g. age of patient and revision of a joint arthroplasty). Adjusted analysis explores the prognostic association of a prognostic factor and an outcome after accounting for (i.e. adjusting for) the prognostic value of other (typically existing and well-established) prognostic factors, using statistical modelling methods such as multivariable regression. Relying solely on unadjusted analysis is rarely sufficient to inform clinical practice because prognostic factors are unlikely to be independently associated with an outcome when other factors are taken into account.^5^ For example, conducting an unadjusted analysis to explore the association between the type of hip arthroplasty surgery and mortality without adjusting for key variables such as the indication for surgery, patient age, and comorbidities may lead to a misleading conclusion. One might find that hip hemiarthroplasty is associated with higher mortality compared with total hip arthroplasty, but this result is of little clinical value when in truth mortality is associated with a range of prognostic factors and the association with type of surgery offers no extra prognostic information over and above the other factors. It is therefore important to understand the prognostic relationship of a factor (such as the type of surgery performed) with the outcome (such as death) once other known factors have been accounted for (such as indication for surgery, patient age, and comorbidities).^30^

A good understanding of the outcome data of interest is another important requirement, so that an appropriate statistical model is used (e.g. logistic regression for a binary outcome with a relatively short time window, Cox proportional hazards regression for a time-to-event outcome).^29^ Performing a sample size calculation, as carried out in research in other fields, is an important consideration and the PROGRESS group recommends taking into account the work published by Schmoor et al^31^ which provides considerations for sample size calculations in prognostic factor research using survival analysis methods. This approach to sample size calculation ensures adequate power for multivariable statistical analysis, which is crucial to reach valuable conclusions.

Other statistical recommendations include:

- Ensure factors are measured at or before the intended moment of prognostication.
- Analyze continuous factors on their continuous scale (avoiding dichotomization or categorization) and ideally examine non-linear trends using fractional polynomials or splines.^5,29^
- Establish the cause of missing data and, where appropriate, consider using methods such as multiple imputation to handle missing data.^5,29^
- Undertake sensitivity analyses to assess the impact of assumptions made.^5^
- Consider that a prognostic factor can relate to longitudinal information, not just be a static value.^32^ For example, surgeon or hospital volume.

### Reporting guidelines

Transparent reporting is a fundamental part of good research and researchers should report all prognostic factors investigated clearly and avoid reporting only those with statistically significant associations with the outcome.^25^ Transparent reporting is essential to differentiate high-quality from low-quality evidence and to reduce publication and reporting biases, facilitating systematic reviews and meta-analyses.^25^ The reporting recommendations for tumour MARKer prognostic studies (REMARK) are recommended for use in prognostic factor research (not just in tumour marker prognostic studies).^25^

### Risk of bias assessment

Another valuable resource is the Quality In Prognosis Studies (QUIPS) tool, specifically developed to assess the risk of bias in prognostic factor studies across six key domains: study participation, attrition, prognostic factor measurement, outcome measurement, adjustment for other prognostic factors, and statistical analysis/reporting.^24^

It can be useful using the QUIPS tool early in the research process, as it helps researchers systematically identify and address potential sources of bias when designing their study protocol. This thorough approach enhances the methodological rigour and validity of prognostic research, ultimately improving the reliability and applicability of the study findings.^24^

In conclusion, prognostic factor research has many purposes in T&O, including informing individual outcome risks, patient counselling, treatment choice, service planning, prediction model development, and designing further research. Prognostic factor research, however, would benefit from prospective, protocol-driven, confirmatory studies to determine new prognostic factors that add value over established prognostic factors and to move away from exploratory research to a greater focus on confirmation and replication. The published recommendations and guidance regarding study design, conduct, analysis, and reporting of prognostic factors studies should be used by researchers, funding bodies, journal editors, and reviewers if we are to derive the maximum benefit for our discipline and ultimately for our patients.^5^

**Take home message**

- Prognostic factor research is central to personalized orthopaedic care, as it provides information that helps inform patients about their condition and supports shared decision-making tailored to individual risk profiles.

- Improving methodological quality and reporting standards is important, as poorly designed or analyzed studies can lead to misleading conclusions. Adopting robust study designs, appropriate statistical methods, and transparent reporting enhances the reliability and clinical relevance of research findings.

## Data Availability

This study did not involve the use of any primary or secondary data. Therefore, no datasets were generated, analyzed, or shared as part of this research.
